# Oral administration of phytochemicals protects honey bees against cognitive and motor impairments induced by the insecticide fipronil

**DOI:** 10.1371/journal.pone.0300899

**Published:** 2024-03-25

**Authors:** Lina M. García, Valentina Caicedo-Garzón, Andre J. Riveros

**Affiliations:** 1 Departamento de Biología, Facultad de Ciencias Naturales, Universidad del Rosario, Bogotá, Colombia; 2 Department of Neuroscience, College of Science, University of Arizona, Tucson, Arizona, United States of America; University of Alberta, CANADA

## Abstract

Pollution produced by exposure to pesticides is a major concern for food security because the negative impacts on pollinators. Fipronil, an insecticide broadly used around the globe has been associated with the ongoing decline of bees. With a characteristic neuroactive toxicodynamic, fipronil leads to cognitive and motor impairments at sublethal dosages. Despite of regional bans, multilevel strategies are necessary for the protection of pollinators. Recent evidence suggests that specific nutrients in the diets of bees may induce protection against insecticides. Here, we evaluated whether the administration of three phytochemicals, namely rutin, kaempferol and p-coumaric acid provide protection to the Africanized honey bee *Apis mellifera* against oral administration of realistic dosages of fipronil. We tested the potential impairment produced by fipronil and the protection induced by the phytochemicals in learning, 24h memory, sucrose sensitivity and motor control. We found that the administration of fipronil induced a concentration-dependent impairment in learning and motor control, but not 24h memory or sucrose sensitivity across a 24h window. We also found that the administration of rutin, p-coumaric acid, kaempferol and the mixture was innocuous and generally offered protection against the impairments induced by fipronil. Overall, our results indicate that bees can be prophylactically protected against insecticides via nutrition, providing an alternative to the ongoing conflict between the use of insecticides and the decline of pollinators. As the studied phytochemicals are broadly present in nectar and pollen, our results suggest that the nutritional composition, and not only its production, should be considered when implementing strategies of conservation via gardens and co-cropping.

## Introduction

Pesticide pollution is a major concern due to its impact on beneficial species and public health. For example, bees are central to crop pollination and food security [1], yet endure individual and colony challenges due to sublethal exposures to pesticides used for pest control [2]. Among sublethal effects, impairment of cognitive functions is particularly critical because of its cryptic yet deteriorating impact [3]. Following robust evidence, certain neuroactive pesticides, including neonicotinoids and fipronil (a phenylpyrazole) were first partially and then permanently banned in the European Union [4]. However, the accelerated decline of managed and wild populations and the timelines for the development and implementation of regulatory policies demand urgent complementary strategies.

In this context, the idea of reducing the impact of pesticides and protecting bees through dietary supplementation with phytochemicals has emerged as an alternative [5]. For example, p-coumaric acid extends the longevity of honey bees [6–8], and gallic acid, abscisic acid, and quercetin improve pathogen tolerance, immunocompetence, and antioxidant activity [6, 9]. Also, some phytochemicals present in pollen improve the tolerance of bees to pesticides [10] and upregulate gene expression for cytochrome P450, enhancing detoxification [11]. Similarly, quercetin and p-coumaric acid increase the survival of honey bees exposed to pesticides [7, 10]. Moreover, the prophylactic administration of phytochemicals such as rutin, kaempferol, and p-coumaric acid are investigated as alternative treatments for human neurological disorders [12, 13].

Thus, nutrition with phytochemicals that are readily available to bees in pollen and nectar seems an important component positively influencing the physiology of bees that are exposed to pesticides. However, supplementation with phytochemicals in bees requires more evidence in the context of protection against cognitive impairments. We have found that the flavonoids rutin and quercetin are associated with the protection of the Africanized honey bees against oral administration of imidacloprid, a first-generation neonicotinoid (LM. Garcia, JJ. Sutachan, CF. Morantes-Ariza, V. Caicedo-Garzón, SL. Albarracin, AJ. Riveros, unpublished). Recently, we also found that the administration of rutin protects the performance of bumble bees against impairment by imidacloprid and fipronil [14]. These results open new questions on the scope of protection against pesticides with different toxicodynamic and the potential of other plant-derived secondary metabolites as neuroprotectors of bees. Here, we tackle these questions by testing the protection offered by three phytochemicals, namely rutin, p-coumaric acid, and kaempferol, against the impairments induced by the oral administration of fipronil, a broadly used insecticide.

Our goal was twofold. First, we aimed to evaluate the potential impairment of a sublethal exposure to a commercial form of fipronil on learning and memory, latency of a learned response, sucrose sensitivity, and motor activity. We focused on fipronil because sublethal doses affect the use of energy [15] and the reproductive [16], motor [17], and cognitive performances [18]. Pharmacologically, fipronil acts by inhibiting the GABAergic control (an inhibitory system itself), leading to excitation of neuronal activity [19]. Also, fipronil negatively impacts the mushroom bodies, major regions of multimodal integration within the central brain of insects [20], and the central complex, a region recognized for its role in navigation and motor control [21, 22]. Hence, exposure to fipronil ultimately impacts foraging by the individual and the colony. Lastly, by selecting a commercial form of fipronil, we aimed to test a realistic scenario for impairment and, eventually, protection.

Second, we aimed to test whether the administration of rutin, kaempferol and p-coumaric acid, separately and in combination, prevents cognitive and motor impairments induced by fipronil. Importantly, these phytochemicals found in pollen and nectar can activate signaling pathways for neuronal survival, synaptic plasticity, and detoxification [23–25]. However, their protective effect on cognitive abilities has not been tested in honey bees, separately or combined, (but see [26, 27]). Importantly, since bees may be regularly exposed to these molecules through the pollen and nectar collected, our results highlight the potential to use plant-derived molecules as nutraceutical agents for pollinators, and the importance of high diversity of plants. Moreover, our results may help improving conservation strategies through identification of floral resources rich in specific metabolites.

## Results

### Determination of dosage of fipronil

**Fig 1:**
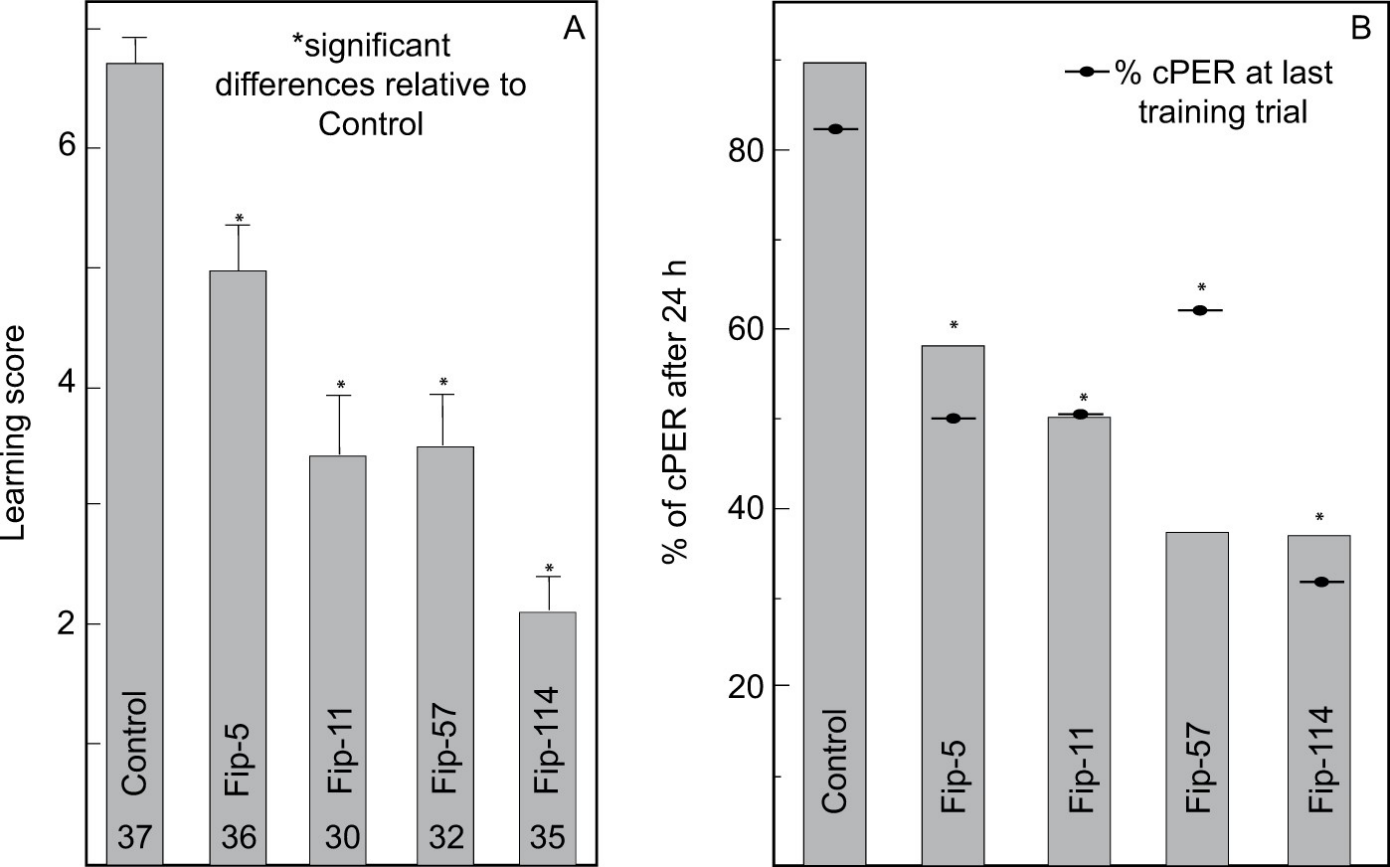
Effect of concentration of fipronil on acquisition and retention of an olfactory stimulus. (**A**) Learning score as the summatory of cPER across training trials. (**B**) Retention as percentage of cPER after 24h. Statistical comparisons were conducted between performance at the last training trial (as indicated by symbol) and performance at 24h (as indicated by the bars). Percentages of cPER at the last training trial are presented for comparison. Sample sizes are indicated within each bar. Learning score bars display standard error of the mean. Statistically significant planned comparisons with respect to the control group are indicated (*). Doses used for this experiment were as follow: Control (1M sucrose water), Fip: 0.04ng/bee (Fip-5). 0.1ng/bee (Fip-11), 0.5ng/bee (Fip-57) and 1ng/bee (Fip-114).

We found that the administration of fipronil impaired the performance of bees (Kruskal-Wallis’ test: χ^2^_4_ = 68.82, P < 0.0001; Fig 1A). Bees in all the groups exposed to fipronil exhibited learning scores significantly lower than Control bees (Steel method: Fip-5: z = -3.51, P = 0.0017; Fip-11: z = -5.13, P = 0.0001; Fip-57: z = -5.87, P = 0.0001; Fip-114: z = -6.84, P = 0.0001). Also, the latency of cPER was significantly impacted by the administration of fipronil and differed between bees across treatments (Kruskal-Wallis’ test: χ^2^_4_ = 20.20, P = 0.0005). Bees in the Fip-5, Fip-57 and Fip-114 groups exhibited significantly longer latencies than Control bees (Dunnett’s method: Fip-5: LSD = 0.04, P = 0.040; Fip-57: LSD = 0.27, P = 0.002; Fip-114: LSD = 0.42, P < 0.0001). In contrast, bees in the Fip-11 group exhibited latencies that were not significantly different from Control (Dunnett’s method: Fip-14: LSD = -0.12, P = 0.152).

After 24 h, the percentage of cPER relative to the last training trial was not significantly different within each treatment (Fischer’s exact test for the comparison between last training trial and 24h test: Control: P = 0.84, Fip-5: P = 0.99, Fip-11: P = 0.98, Fip-57: P = 0.99, Fip-114: P = 0.98; Fig 1B), indicating that memory retention was not affected. Nevertheless, the differences across treatments observed during acquisition were maintained after 24h (Likelihood ratio: χ^2^ = 29.40, P < 0.0001; Fig 1B) with a dose-dependent decrease in percentage of bees that exhibited a conditioned PER: Control (89.1%), Fip-5 (58.3%), Fip-11 (50%), Fip-57 (37.5%), Fip-114 (37.1%). In all groups receiving fipronil, bees exhibited a probability of a conditioned PER that was significantly lower than the observed in Control bees (Adjusted Wald test for two proportions relative to Control group: Fip-5: 0.31, P = 0.0012; Fip-11: 0.39, P = 0.0002; Fip-57 = 0.51, P = 0.0002; Fip-114 = 0.52, P = 0.0002). Similarly, the odds of remembering were estimated to be always higher for Control bees and dose dependent: 5.8 times relative to Fip-5 bees (95% CI: 1.72–20.18), 8.2 times relative to Fip-11 bees (95% CI: 2.33–29.10), 13.7 times relative to Fip-57 bees (95% CI: 3.89–48.5), and 13.9 times relative to Fip-114 bees (95% CI: 4.02–48.42).

### Evaluation of the protective effect of the prophylactic administration of phytochemicals

#### Olfactory conditioning of the proboscis extension response

**Fig 2:**
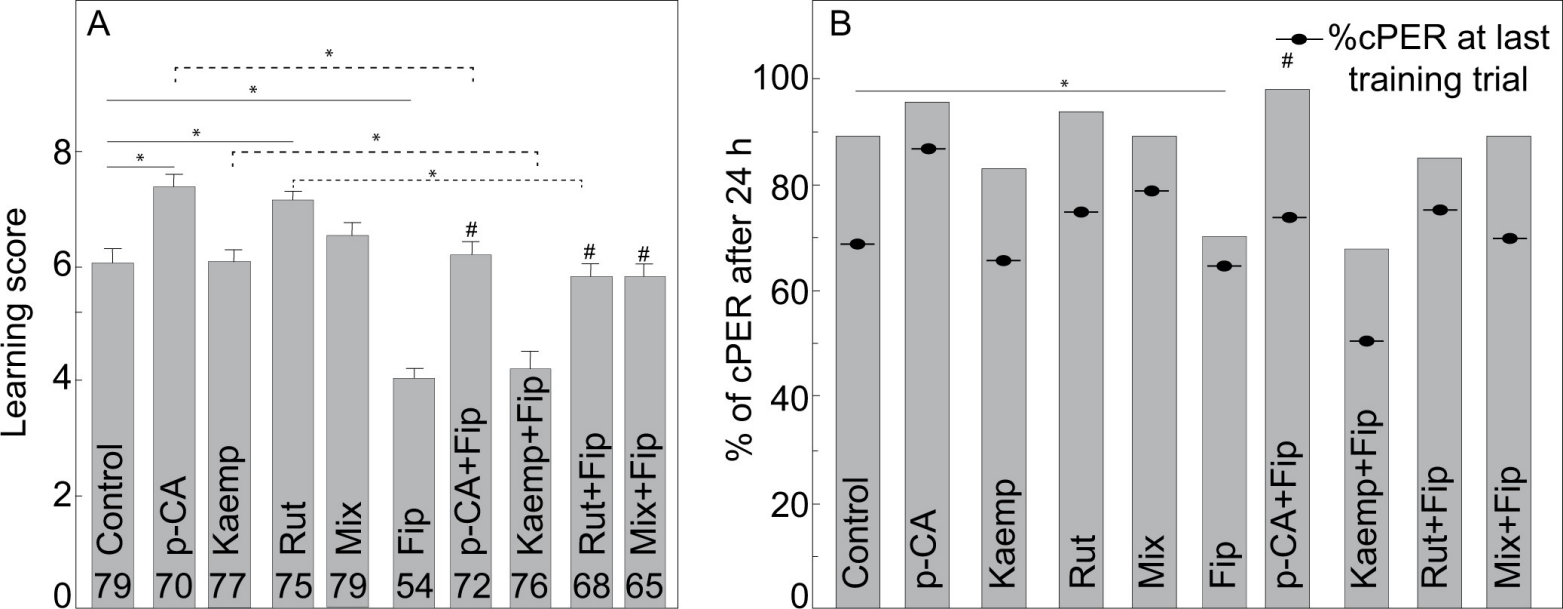
Effect of the administration of p-coumaric acid (p-CA), kaempferol (Kaemp), rutin (Rut) and their combination (Mix), and fipronil (Fip) on acquisition and retention of an olfactory stimulus. (**A**) Learning score as the summatory of cPER across training trials (**B**) Retention as percentage of cPER after 24h. Statistical comparisons were conducted between performance at the last training trial (as indicated by symbol) and performance at 24h (as indicated by the bars). Percentages of cPER at the last training trial are presented for comparison. Sample sizes are indicated within each bar. Learning score bars display standard error of the mean. Statistically significant planned comparisons with respect to the respective control group are indicated (*). Statistically significant planned comparisons with respect to the fipronil group are indicated (#). Doses used for this experiment were as follow: Control (1M sucrose water), p-CA (328ng/bee), Kaemp (374.9ng/bee), Rut (6ng/bee), Mix (328ng/bee of p-CA + 374.9 ng/bee of Kaemp + 6ng/bee of Rut), Fip: 1ng/bee.

Subsequent experiments were conducted using a dose of 1 ng/bee of fipronil as it significantly impaired learning acquisition and maintained the impairment after 24 h without entirely abolishing the performance (Fig 2).

We found that the learning score significantly varied between bees exposed to different feeding treatments (mean score ± s.e.m: Control: 6.0±0.26, Rut = 7.1±0.21, Kaemp = 6.1±0.21, p-CA = 7.4±0.20, Mix = 6.5±0.20, Fip = 4±0.12, Rut+Fip = 5.8±0.26, Kaemp+Fip = 4.1±0.3, p-CA+Fip = 6.2±0.2, Mix+Fip = 5.8±0.2. Kruskal-Wallis’ test: χ^2^_9_ = 144.23, P < 0.0001; Fig 2A). The performance of bees fed with all the phytochemicals did not significantly differ from Control bees (Steel method test: Kaemp: z = -0.070, P = 1.0; Mix: z = -1.30, P = 0.80) or was significantly enhanced (Steel method test: p-CA: z = 3.64, P = 0.003; Rut: z = 2.93, P = 0.037). In contrast, the administration of 1 ng/bee of fipronil impaired learning and the bees in the Fip group exhibited significantly lower learning scores than bees in the Control group (Steel method test for Fip vs. Control: Fip: z = -5.37, P = 0.0003).

Most importantly, prophylactic administration of most of the molecules or the mix offered full or partial protective effect. Generally, bees fed with a molecule and then with the insecticide exhibited significantly higher scores than bees fed only with fipronil (Steel method test: Rut+Fip vs. Fip: z = 4.92, P = 0.0003; p-CA+Fip vs. Fip: z = 6.83, P = 0.0003; Mix+Fip vs. Fip: z = 6.12, P = 0.0003), indicating successful protection. An exception to this was observed in the bees fed with kaempferol (Kaemp+Fip vs. Fip: z = 1.44, P = 0.67). However, bees fed with a molecule and then with the insecticide generally exhibited learning scores that were significantly lower than bees fed only with their respective control molecule (Steel method test: Rut+Fip vs. Rut: z = -3.66, P = 0.003; Kaemp+Fip vs. Kaemp: z = -4.25, P = 0.0005; p-CA+Fip vs. p-CA: z = -3.90, P = 0.0017; Fig 2). An exception occurred in bees fed with the mix and then with the pesticide, which exhibited learning scores that did not significantly differ from bees fed only with the mix (Steel method test: Mix+Fip vs. Mix: z = -2.52, P = 0.103). Thus, the administration of rutin and p-coumaric acid offered partial protection whereas the administration of the mix offered full protection.

Also, we found that the administration of the treatment significantly affected the latency of response (Kruskal-Wallis’ test: χ^2^_9_ = 28.63, P = 0.001). In most cases, bees across groups exhibited latencies that did not significantly differ from their respective control bees (Steel method: Fip vs. Control: z = 0.58, P = 1.0; Rut vs. Control: z = -0.25, P = 1.0; Kaemp vs. Control: z = 2.21, P = 0.88; p-CA vs. Control: z = 1.44, P = 1.0; Mix vs. Control: z = 1.07, P = 1.0; Rut+Fip vs. Rut: z = 0.42, P = 1.0; p-CA+Fip vs. p-CA: z = 0.0, P = 1.0; Mix+Fip vs. Mix: z = 1.98, P = 0.88; Kaemp+Fip vs. Kaemp: z = 1.53, P = 1.0). Also, the latency of response did not differ significantly between the bees prophylactically administered with the compositions and the bees receiving fipronil (Steel method: Rut+Fip vs. Fip: z = -0.36, P = 1.0; p-CA+Fip vs. Fip: z = 0.70, P = 1.0; Mix+Fip vs. Fip: z = 2.04, P = 0.88; Kaemp+Fip vs. Fip: z = 2.57, P = 0.80).

After 24 h, we did not observe a significant difference in percentage of cPERs within each treatment relative to the last training trial (Fischer’s exact test: Control: P = 0.47; Fip: P = 0.76; p-CA: P = 0.98; Rut: P = 0.89; Kaemp: P = 0.99, Mix: P = 0.24; Rut+Fip: P = 0.99; p-CA+Fip: P = 0.98; Kaemp+Fip: P = 1.0; Mix+Fip: P = 0.96). Nevertheless, the probability of exhibiting a cPER was significantly different across treatment (Likelihood ratio: χ^2^ = 37.59, P < 0.0001; Fig 2B). The bees fed with phytochemicals exhibited a percentage of cPER that did not significantly differ from the bees in the Control group (Adjusted Wald test for two proportions: Rut vs. Control: 0.045, P = 0.55; p-CA vs. Control: 0.066, P = 0.44; Kaemp vs. Control: 0.055, P = 0.55; Mix vs. Control: 0.00, P = 1.0). In contrast, the administration of fipronil decreased the probability of exhibiting a cPER relative to Control bees but the difference was barely statistically significant (Adjusted Wald test for two proportions-Control vs. Fip: -0.181, P = 0.05). Importantly, the bees that received the prophylactic administration of most of the phytochemicals had a probability of exhibiting a cPER that did not significantly differ from their respective control bees (Adjusted Wald test for two proportions: Rut+Fip vs. Rut: 0.08, P = 0.36; Mix+Fip vs. Mix: 0.001, P = 1.0; p-CA+Fip vs. p-CA: -0.01, P = 0.86; Kaemp+Fip vs. Kaemp: 0.14, P = 0.113) but in one case was significantly higher than the probability of a cPER in the bees exposed to fipronil (Adjusted Wald test for two proportions: p-CA+Fip vs. Fip: 0.26, P = 0.0013). Also resembling the results observed during acquisition, the bees in some groups had a probability of exhibiting a cPER that did not significantly differ from Fip bees (Adjusted Wald test for two proportions: Kaemp+Fip vs. Fip: 0.02, P = 0.55; Mix+Fip vs. Fip: 0.17, P = 0.05; Rut+Fip vs. Fip: 0.14, P = 0.11).

#### Climbing assay

**Fig 3:**
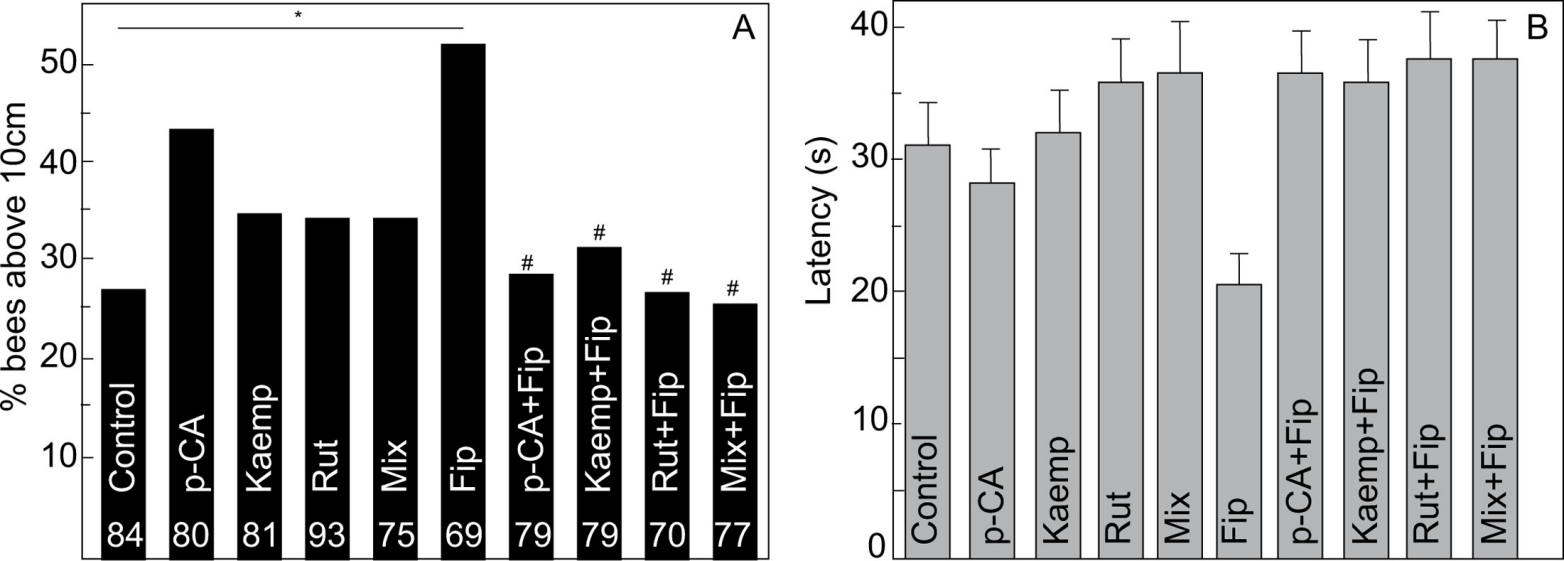
Effect of the administration of phytochemicals and fipronil on motor performance during a climbing assay. (**A**) Percentage of bees that crossed the 10 cm threshold. (**B**) Time (displayed as mean±s.e.m) to reach the threshold. A and B include the same individuals. Statistically significant planned comparisons are indicated with respect to bees in the Control group (*) and relative to bees in the Fip group (#). Only significant comparisons with *P<0.05 or # P<0.05 and indicated for better visualization. Doses used for this experiment were as follow: Control (1M sucrose water), p-CA (328ng/bee), Kaemp (374.9ng/bee), Rut (6ng/bee), Mix (328ng/bee of p-CA + 374.9 ng/bee of Kaemp + 6ng/bee of Rut), Fip: 1ng/bee.

We found that the ability to climb was not significantly affected by the feeding treatments (Likelihood ratio: χ^2^: 15.54, P = 0.08; Fig 3). Nevertheless, we pursued the planned comparisons motivated by the visual differences indicated by plotting the data (Fig 3). Interestingly, we found that the bees receiving fipronil tended to climb above the threshold significantly more often than bees in the Control group (Adjusted Wald test for two proportions-Control vs. Fip: 0.21 P = 0.02, Fig 3A). Also, the average speed of climbing of the bees that received fipronil was higher yet did not significantly differ from the speed of bees in the Control group (Steel method: mean speed±s.e.m. = Fipronil: 0.98±0.10 cm s^-1^, Control: 0.56±0.05 cm s^-1^; Fip vs. Control: z = 2.92, P = 0.067; Fig 3B).

In contrast, the bees fed with any of the phytochemicals did not exhibit significant differences in the number of bees reaching the threshold relative to individuals in the Control group (Adjusted Wald test for two proportions: Control vs. Kaemp: 0.027, P = 0.76; Control vs. Rut: 0.053, P = 0.55; Control vs. p-CA: 0.123, P = 0.19; Control vs. Mix: 0.053, P = 0.55; Fig 3) or in their speed relative to bees in the Control group (Kaemp: mean speed±s.e.m. = 0.54±0.05 cm s^-1^, z = -0.46, P = 0.99; Rut: mean speed±s.e.m. = 0.54±0.04 cm s^-1^, z = -0.58, P = 0.99; p-CA: mean speed±s.e.m. = 0.64±0.05 cm s^-1^, z = 0.90, P = 0.99; Mix: mean speed±s.e.m. = 0.52±0.05 cm s^-1^, z = -0.92, P = 0.99; Fig 3).

Importantly, the prophylactic administration of phytochemicals generally conferred a protective effect against the impairment induced by fipronil. The percentage of bees reaching the threshold did not significantly differ between bees receiving a phytochemical and the bees that received it and were subsequently exposed to fipronil (Adjusted Wald test for two proportions: Rut vs. Rut+Fip: 0.06, P = 0.55; Kaemp vs. Kaemp+Fip: 0.02, P = 0.76; p-CA vs. p-CA+Fip: 0.12, P = 0.19 and Mix vs. Mix + Fip: 0.1, P = 0.31; Fig 3A). This protective effect was further confirmed by the significant decrease to normality in the percentage of bees reaching the threshold compared to the bees that received only Fip (Adjusted Wald test for two proportions: Rut+Fip: 0.22, P = 0.02; Kaemp+Fip: 0.20, P = 0.02; p-CA+Fip: 0.21, P = 0.02 and Mix+Fip: 0.252, P = 0.011).

Lastly, the effective protection by phytochemicals was observed when measuring climbing speed. Relative to bees in the Fip group, prophylactically protected bees exhibited a significant reduction in speed (Kaemp+Fip: mean speed±s.e.m. = 0.47±0.04 cm s^-1^, z = -3.93, P = 0.003; Rut+Fip: mean speed±s.e.m. = 0.47±0.04 cm s^-1^, z = -3.86, P = 0.003; p-CA+Fip: mean speed±s.e.m. = 0.58±0.06 cm s^-1^, z = -3.63, P = 0.007 and Mix+Fip: mean speed±s.e.m. = 0.49±0.05 cm s^-1^, z = -4.15, P = 0.003); yet, the speed of protected bees did significantly differ from bees in their respective control groups (see speed for each group above; Rut vs. Rut+Fip: z = -0.56, P = 0.99; Kaemp vs. Kaemp+Fip: z = -0.69, P = 0.99; p-CA vs. p-CA+Fip: z = -1.85, P = 0.706; Mix vs Mix+Fip: z = -0.59, P = 0.99).

**Sucrose sensitivity assay.** Overall, we did not find any effect of the feeding treatments on the total score of bees (mean score±s.e.m.: Control = 20.6±1.4; Rut = 17.9±1.7; Kaemp = 19.3±1.4; p-CA = 18.1±1.7, Mix = 22.1±1.2; Fip = 20.3±1.3; Rut+Fip = 17.3±1.8; Kaemp + Fip = 18.1±1.6; p-CA+Fip = 19.8±1.5; Mix+Fip = 19.6±1.4; Kruskal-Wallis test: χ^2^_9_ = 6.02, P = 0.74). Our more detailed comparisons relative to sensitivity exhibited by the bees in the Control group indicated that there were not significant deviations in the sensitivity to sucrose during the 3h, 5h or 27h (P > 0.05 in all comparisons). Nevertheless, we observed a slight increase in sensitivity in bees receiving the Mix of the phytochemicals, yet it rendered non-significant from the Control bees.

## Discussion

The pollution produced by insecticides is a growing concern around the globe. Paradoxically, insecticides are central tools for crop production, yet their impact on pollinators and other beneficial species may compromise productivity. While extant regulations may contribute to ameliorate the problem, multilevel approaches need to be implemented. Here, we aimed to evaluate the potential of prophylactic administration of phytochemicals for protecting Africanized honey bees against impairments produced by oral administration of fipronil, an insecticide commonly used in many countries. Our approach produced three key results. First, the oral administration of fipronil led to a dose-dependent impairment of learning and motor control, but not of memory retention or sucrose sensitivity. Second, the administration of the phytochemicals was innocuous when administered separate or as a mix. Third, the prophylactic administration of the phytochemicals generally, but not always, led to partial or full amelioration of the impairments produced by the fipronil.

The impairment in learning acquisition and motor control observed in our bees administered with fipronil may be explained by its toxicodynamic [28]. Fipronil acts as an antagonist of the GABA_A_R [29] and the glutamate-gated chloride channels [19, 30, 31], consequently blocking inward chloride currents that are key for inhibitory synapses [32]. Moreover, the exposure to fipronil may lead to neurodegenerative processes [33]. Hence, it is not surprising that fipronil impaired learning acquisition, and motor control, given the abundance of GABAergic pathways in brain centers involved in such processes (e.g., antennal lobes, the mushroom bodies, and the central complex [32, 34]). Thus, lower olfactory acquisition may derive from impairment of the olfactory coding [35, 36]. Interestingly, the impairment observed here differed from previous accounts in two directions. On the one hand, we did not observe an impact on memory retention, inferred from the maintained level of conditioned responses between the last training trial and the 24h test (Fig 2B). Whereas previous accounts (e.g., [18, 36]) have reported impairment of memory, the comparisons have been made relative to the retention of bees in control groups, which does not consider the variation from the last training trial and the testing event within a group. We argue that the low percentage of conditioned PER recorded after 24h derived from poor acquisition and not necessarily from impaired storage capacity (see also [14]). Similarly, we observed a dose-dependent impairment: the administration of the increasing dosages from 0.04ng/bee to 0.1ng/bee, 0.5ng/bee and 1ng/bee led to decreases of 35%, 43.9%, 57.9% and 58.4%, respectively, relative to bees in the Control group (see also Fig 1B). Previous accounts have shown that higher dosages of fipronil (including the one used here) may lead to lower impacts [35, 36]. These differences might derive from interspecific and geographic variations in sensitivity to pesticides (Africanized vs. European honey bee; e.g., [37, 38]), the administration of commercial vs. pure fipronil, and the time of exposure to the pesticide. Also, our results are in agreement with previous observations that the oral administration of fipronil does not impair the sensitivity to sucrose [18], suggesting that the effects observed here in learning acquisition derived from impacts on higher centers within the brain.

Importantly, a commonly reported effect of fipronil intoxication is an impaired motor control. The administration of fipronil caused an increase in walking speed. This result likely derives from the hyperexcitability and overstimulation of the nervous system byproduct of the blockage of the inhibitory pathways [30]. We recently observed a similar impact in the fruit fly *Drosophila melanogaster* after inducing a so-called parkinsonian phenotype following the oral administration of fipronil [39]. In this case, besides the mushroom bodies, the behavior observed may reflect the impact in the central complex, a GABA_A_R-rich brain region long recognized for its role in motor control [40]. Moreover, effective locomotion also requires visual information, which was likely impaired in the fruit fly through dopaminergic signaling [39]. Whether fipronil also impacted visual processing in our bees was not evaluated but requires further study because in a real scenario it might impair flight control.

More importantly, the phenolic acids were innocuous and generally protected the performance of the bees during the cognitive and motor tasks. On the one hand, the innocuity of the individual molecules and the mix likely derived from their presence in pollen and nectar and the low dosages used [6, 10, 41]. On the other hand, given the effects of fipronil, the cognitive protection conferred by the administration of the phytochemicals may derive from structural and/or physiological effects. Our behavioral level of analysis does not allow us to define a mechanistic explanatory framework. However, below we introduce four potential scenarios based on our current knowledge of the effect of some of the phytochemicals used. Testing these scenarios requires methods beyond behavioral analyses but could enrich our understanding of the mechanisms of protection.

First, phytochemicals may support protection via enhancement and maintenance of structural integrity. For instance, rutin modulates neuronal plasticity (e.g., growth of dendritic branches) and cell viability in vitro [42, 43]. Whether this regulation occurs within the mushroom bodies and in the central complex of bees would require analyses of the expression of the molecules and cascades known to be involved (e.g., protein kinase 1 ERK1/2, MAPK the mitogen-activated protein kinase (MAPK) cascade [42] the expression of the nuclear factor CREB [44]).

Second, phytochemicals may protect via a reduction of neurodegeneration. As other neuroactive pesticides, the impact of fipronil extends beyond the membrane activity, destabilizing mitochondrial function and eventually triggering apoptosis [28]). Mitochondrial protection may include the regulation of the oxidative stress induced by fipronil [45]. Phenolic acids contribute to the reduction of oxidative stress (reviewed by [46, 47]). In this direction, the phytochemicals may increase the activity level of intracellular antioxidant enzymes to stop the production of free radicals and ROS. Moreover, phenolic acids such as kaempferol, rutin, and p-coumaric acid inhibit the Nf-Kb signaling pathway, preventing neurotoxicity [48]. Finally, in rats, prophylactic administration of kaempferol decreases hippocampal neurodegeneration induced by the insecticide chlorpyrifos [49]. Similarly, p-coumaric acid reduces dopaminergic neurodegeneration and mitigates motor deficits in mice exposed to the insecticide rotenone [50].

Third, phytochemicals may interfere with the action of fipronil or favor its metabolization. On the one hand, we have shown that, in fruit flies, the administration of kaempferol protects against motor impairments produced by fipronil [39], and bioinformatic analyses suggest an allosteric regulation of the GABA_A_R. On the other hand, kaempferol, rutin, and p-coumaric acid upregulate CYP450 enzymes, favoring insecticide metabolization [51, 52]. Interestingly, hydroxyphenylacetic acid (4-HPAA; [53–55], a metabolite of kaempferol and endogenous dopamine, activates enzymes, such as CYP450, involved in degrading xenobiotic molecules [56].

Interestingly, we observed a positive synergy between phytochemicals as predicted for the bees in the Mix treatment. This suggests that the protection offered by one molecule does not interfere with protection offered by the other molecules nor produces negative effects. We hypothesize a more effective protection derived from a convergent regulation of GABA_A_R, probably in multiple binding sites [39, 57–59]. As we observed full protection following administration of the Mix, we suggest that binding sites for our molecules in the GABA_A_R might be different and regulate together the receptor.

We conclude that rutin, kaempferol and p-coumaric acid are most generally effective separated and as a mix to protect against impairments produced by oral administration of fipronil in Africanized honey bees. Our findings add evidence to the enormous potential of phenolic acids and other secondary metabolites derived from plants as an option to protect bees in environments polluted with insecticides. Whereas this approach may prove more effective for managed bees, the broad and extended variation of phenolic acids in pollen and nectar suggest that urban gardens may be planned with better precision and that increase biodiversity in agroecosystems may be beneficial for bees and other pollinators.

## Materials and methods

### Collection and maintenance of experimental subjects

We collected workers of the Africanized honey bee *Apis mellifera* leaving one of the hives at the Apiary of the Universidad del Rosario (Bogota, Colombia) between 0800h and 1000h. The hive had 10 brood combs and the queen bee was 3 to 4 weeks old age. Once in the lab, the bees were cold anesthetized by keeping them on a plastic container on ice and restrained in plastic tubes such that their heads were held in place and the bees could extend their proboscis. The bees were kept under the natural conditions of the laboratory (RH: 62%; T: 19°C) for the duration of the experiment. Bees were fed and maintained according to the assigned treatment.

### Determination of dosage of fipronil

We collected and maintained 172 bees. We excluded two bees (one for Control and one for fip-57) after screening for outliers. Thus, we conducted our final analyses using 170 bees distributed across five treatments (Fig 1): Control (N = 37), Fip-5 (N = 36), Fip-11 (N = 30), Fip-57 (N = 32) and Fip-114 (N = 35).

### Protective effect of the prophylactic administration of phytochemicals

#### Olfactory conditioning of the proboscis extension response

We collected and maintained 858 bees. We excluded 143 bees after an outlier screening (Control = 14 bees, Rut = 14, Kaemp = 9, p-CA = 17, Mix = 10, Fip = 36, Rut+Fip = 11, p-CA+Fip = 12, Mix+Fip = 20). Thus, for the final analyses we included 715 bees across 10 treatments (Fig 2): Control (N = 79), Rut (N = 75), Kaemp (N = 77), p-CA (N = 70), Mix (N = 79), Fip (N = 54), Rut+Fip (N = 68), Kaemp+Fip (N = 76), p-CA+Fip (N = 72), Mix+Fip (N = 65).

#### Climbing assay

We collected and maintained 792 bees. We excluded five bees after an outlier screening (p-CA = 2, Fip = 3). Thus, for the final analyses we included 787 bees across 10 treatments (Fig 3): Control (N = 84), Rut (N = 93), Kaemp (N = 81), p-CA (N = 80), Mix (N = 75), Fip (N = 69), Rut+Fip (N = 70), Kaemp+Fip (N = 79), p-CA+Fip (N = 79), Mix+Fip (N = 77).

#### Sucrose sensitivity assay

We collected and prepared 377 bees distributed across 10 treatments. We excluded 11 bees that died during the experiment. We also excluded 20 bees after an outlier screen analysis. Thus, we conducted our final analyses using 346 bees distributed across ten treatments: Control (N = 35), Rut (N = 36), Kaemp (N = 35), p-CA (N = 34), Mix (N = 34), Fip (N = 37), Rut+Fip (N = 32), Kaemp+Fip (N = 34), p-CA+Fip (N = 34), Mix+Fip (N = 35).

The bees were kept under laboratory conditions (RH: 62% relative humidity; T: 19°C) for the duration of the experiment. Bees were fed and maintained according to the assigned treatment.

### Experimental treatments

#### Determination of dosage of fipronil

First, we determined a concentration of fipronil that would impact performance in learning and memory. We relied on reported realistic doses and times of effect of the pesticide [18, 60, 61] and defined four doses below the reported value: 0.04 ng/bee, 0.1 ng/bee, 0.5 ng/bee, and 1 ng/bee. Each dose corresponded to a treatment. Three hours before olfactory conditioning, bees were randomly allocated to one of these treatments and received a dose of 20 μL of fipronil (Astuto, Invesa S.A.) dissolved using sucrose-water (final concentration for sucrose: 1 M) to the final concentration of the assigned treatment. Thus, bees received one of five treatments: sucrose solution (Control), fipronil 5 nM (Fip-5: 0.04 ng/bee), fipronil 11.43 nM (Fip-11: 0.1 ng/bee), fipronil 57.18 nM (Fip-57: 0.5 ng/bee), fipronil 114.33 nM (Fip-114: 1 ng/bee). To deliver the doses, we stimulated the antennae with 1M sucrose solution to induce a reflexive extension of the proboscis and then fed the pesticide. Thus, the antennae were not contaminated with the pesticide during the administration.

#### Evaluation of the protective effect of the prophylactic administration of phytochemicals

For testing of cognitive performance, we collected and restrained bees as described above, and randomly allocated them to receive one of five feeding treatments, all dissolved to a final sucrose concentration of 1M: sucrose solution (Control, Invitrogen: 15503022), rutin 1 μM (Rut; Sigma-Aldrich: R5143, concentrations according to [14, 42]; kaempferol 131 μM (Kaemp; Sigma-Aldrich: K0133, concentrations adapted from [6], p-coumaric acid 200 μM (p-CA; Sigma-Aldrich: C9008 concentrations following [8], rutin 1 μM + kaempferol 131 μM + p-coumaric acid 200 μM (Mix). In all cases, bees were fed six doses of 10 μL of the composition (two doses a day for three days). On the fourth day, bees were randomly assigned to one of two treatments three hours before training: 10 μL of 1M sucrose solution or 10 μL of fipronil 228.7 nM (Fip; 1ng/bee). Thus, bees belonged to one of ten treatments according to the composition administered: Control, Rut, Kaemp, p-CA, Mix, Fip, Rut+Fip, Kaemp+Fip, p-CA+Fip, Mix+Fip. These treatments were used for the evaluation of protection of learning and memory, motor activity, and sucrose sensitivity.

### Behavioral assays

#### Olfactory conditioning of the proboscis extension response

**Fig 4:**
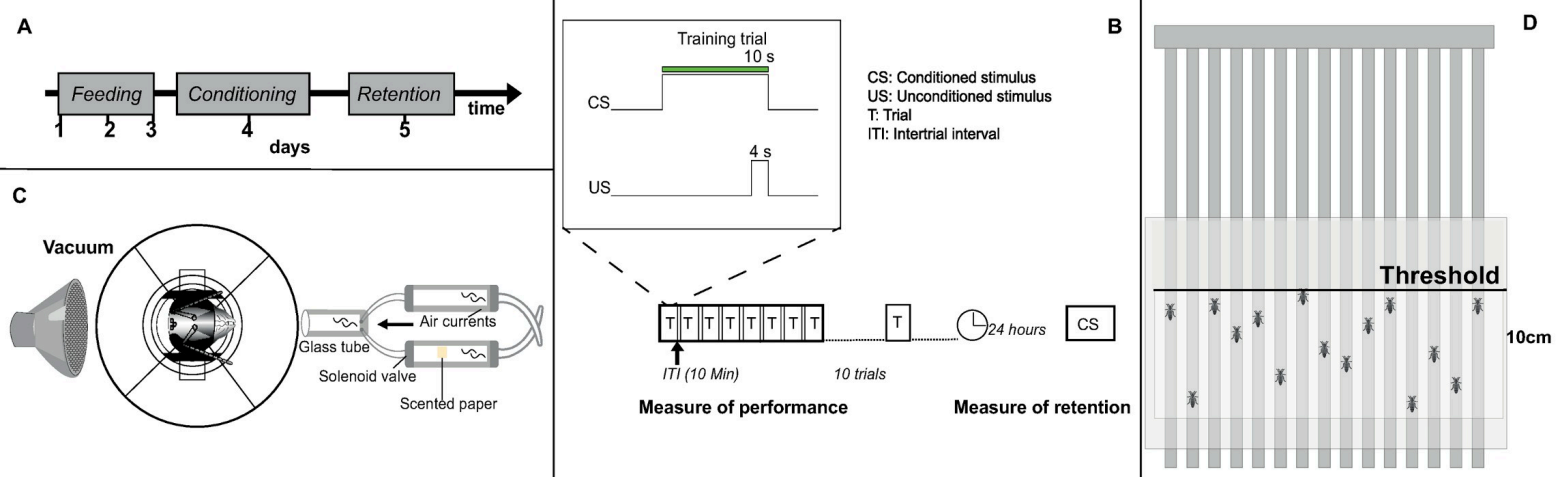
General experimental protocol for administration of feeding treatments, olfactory conditioning, and test of motor control. (**A**) Timeline of the overall protocol. (**B**) Sequence of training trials presentations within a training session and memory retention test. (**C**) Top view of the arrangement for conditioning of a single bee within an individual chamber. (**D**) Vertical arena for the climbing assay. A threshold line was drawn 10cm above the starting point.

Three hours after the administration of treatment, bees were transferred to a rotatory training apparatus holding 12 bees in separate chambers (Fig 4A; after [62]. Bees were allowed to adapt for five minutes before starting a session. To evaluate learning and memory, we relied on standard protocols for the appetitive olfactory conditioning of the proboscis extension response (PER; [63]). Briefly, the bees were trained to learn the association between an olfactory stimulus (the conditioned stimulus, CS) and a reward (the unconditioned stimulus, US). During training, a clean stream of air generated by a pump (air flow: 1.3 L/min) was directed at the bee for 15 s (Fig 4C). Then, the olfactory stimulus was injected for 10 s into the air current. For injection of the stimulus, a second stream of air originating from the same pump was redirected to flow through a tube containing a piece of filter paper with 1 μl of 1-Hexanol (Sigma-Aldrich: 471402). Thus, the air pressure received by the bee was maintained at the same level. Six seconds after the onset of odor delivery, the antennae were stimulated with a solution of 1.5 M sucrose solution. Following the induced PER, the bee was allowed to drink for 4 s. The odor and reward delivery finished simultaneously. This sequence (hereafter a training trial; Fig 4B) was repeated every 10 minutes for eleven times (hereafter a training session) and the conditioned PER (cPER; a PER that follows the stimulation with the odor without antennal stimulation with the sucrose) and the latency of response (time between the onset of odor delivery and the onset of a response that led to a full extension of the proboscis) were recorded. Timing during a training session was monitored with a 2 Hz sound signal from a metronome (Metronome Beats App, Stonekick Limited 2021, London, UK). Once the training session was finished, bees were transferred to a plastic box, fed 10 μL of a 1 M sucrose solution for maintenance until the memory retention test.

Memory retention was tested 24 h after the last conditioning trial by presenting only the olfactory stimulus without reward during a single test. If a bee did not exhibit a conditioned PER, we stimulated the antennae with a drop of a 1 M sucrose solution to test for motivation. Training and testing were conducted by an experimenter blind to treatments. Bees that did not exhibit a PER to the stimulation with the sucrose solution during training or memory test were removed from the experiment.

#### Climbing assay

Three hours after administration of a treatment, we evaluated motor activity. For the test, we used an arena (H: 20 cm, L: 20 cm, W: 0.5 cm; modified from [17]) with 15 lanes (width lane: 0.5 cm) and a visual mark that was used as a threshold (10 cm from the bottom; Fig 4D). One side of the arena was glass-covered, thus allowing the recording of the bees while moving in the lane. For the test, a single bee was gently introduced at the bottom of each lane (at least three bees per treatment could be recorded at the same time using the arena). For each treatment, we recorded the percentage of bees that crossed the threshold in 15 s and the average time for climbing the 10 cm. Thus, we could calculate a speed for each bee. We recorded up to a maximum of 120 s (iPhone 6s, Apple Inc., California). The tests were performed with the arena in a vertical position, thus stimulating locomotion by negative geotaxis.

#### Sucrose sensitivity assay

We assessed responsiveness to sucrose by stimulating the antennae of the bees with ascending concentrations of sucrose [0.1% (w/w), 0.3%, 1%, 3%, 10%, and 30%; [64]]. Bees were collected, restrained in plastic tubes, and fed with 10 μL of a 1 M sucrose solution twice a day for three days. On the fourth day, we measured four times (0 h, 1.5 h, 3 h, and 5 h) the response to sucrose by exposing the bees to the ascending concentrations. Bees were stimulated with pure water before each presentation with the sucrose solution as a control for possible sensitization or habituation. The inter-trial time between the presentation of each concentration of the sucrose solution was on average 4 minutes. A positive response was recorded if the bee exhibited a PER after antennal stimulation with the solution. Thus, for each bee, we obtained a sensitivity score (0–7) that reflected the sensitivity to sucrose at each time interval. Importantly, the first measure (0 h) was conducted prior to the administration of fipronil and was used to normalize the responses across all times. Following the 0 h measure, we randomly assigned the bees to one of the ten treatments described above. After the last measurement (5h), 10 μL of 1 M sucrose solution was fed to the bees for maintenance until the next day for the final measure (27h after pesticide administration). Importantly, the measurements at 3h, 5h and 27h coincide with the period of olfactory conditioning and memory evaluation.

### Statistical analyses

Learning was assessed from the total number of conditioned responses across the ten trials. Thus, each bee received a score between 0–10 (bees should not exhibit a learned response at the first trial). Learning scores were compared across treatments using a Kruskal-Wallis test as data were not normally distributed (evaluated with a Shapiro-Wilk W test). Multiple planned comparisons were conducted comparing learning scores relative to Control bees and fipronil bees using a non-parametric one or two-tailed Steel method depending upon predictions. Based on previous evidence, our predictions were: i. A decreased performance in bees fed with fipronil relative to bees in the Control group, ii. An enhancement of performance in bees prophylactically fed with phytochemicals and then exposed to fipronil relative to bees exposed only to fipronil, and iii. A performance of bees exposed to phytochemicals that would not differ from the performance of bees in the Control group. The following comparisons were of interest because they allow us to test: i. the innocuity of administering phytochemicals and the potential impairment produced by fipronil (Control vs all other groups), ii. the protection induced by the prophylactic treatment ([phytochemicals]+fipronil groups vs. fipronil group), iii. the full or partial protection ([phytochemicals]+fipronil groups vs. [phytochemical] groups). We excluded outliers by using a Mahalanobis distance test [65]. Latency of response was calculated using single or multiple cPERs. We Log transformed the latencies to normalize the distribution (Shapiro-Wilk W test after log transformation: P > 0.05 for all treatments for the null hypothesis that latencies data are from a normal distribution). We run multiple planned comparisons between all treatments and the respective control group (e.g., Rut+Fip vs. Rut, Rut vs. Control) using the Steel method. After 24 hours, the response to the retention test was recorded as a binary response to the sole presentation the conditioned stimulus. Memory was compared across groups using an overall Chi-Square and an Adjusted Wald test for the independent planned comparisons. For motor activity, the percentage of bees that crossed the 10 cm threshold was compared using a Chi-Square test followed by an Adjusted Wald test for the planned comparisons. We compared the speed of the bees using a one or two-tailed Steel method depending upon predictions. For sucrose sensitivity, we conducted comparisons of the total score of bees (0–28 for the sum across times) using a Kruskal-Wallis test. Further, to control for the random initial variation across treatments, we conducted an additional analysis on a normalized score using the sensitivity measured at time 0 h as a baseline [e.g., (score at 3h+1)/(score at 0h+1]. We relied on non-parametric tests using the Steel Method with control to test for deviations from the pattern of change in sucrose sensitivity observed in Control bees across time. In all cases error due to multiple comparisons was controlled using the Classical one stage False Discovery Rate method and adjusted p-values (q-values) are presented [66, 67]. All statistical analyses were conducted using JMP v.15.0 (SAS Institute Inc.).
